# Nevermore: Target-Conditioned Protein–Ligand Representation Learning for Multi-Objective Lead Optimization with Database-Grounded Retrieval

**DOI:** 10.3390/biology15120971

**Published:** 2026-06-21

**Authors:** Mohammad Saleh Refahi, Milad Toutounchian, Bahrad A. Sokhansanj, Hyunwoo Yoo, James R. Brown, Hai-Feng Ji, Gail L. Rosen

**Affiliations:** 1 Department of Electrical and Computer Engineering, Drexel University, Philadelphia, PA 19104, USA; bahrad@molhealtheng.com (B.A.S.); hty23@drexel.edu (H.Y.); jb4633@drexel.edu (J.R.B.); 2 College of Computing & Informatics, Drexel University, Philadelphia, PA 19104, USA; mt3393@drexel.edu; 3 Department of Chemistry, Drexel University, Philadelphia, PA 19104, USA; hj56@drexel.edu

**Keywords:** target-conditioned molecular design, protein–ligand affinity prediction, contrastive representation learning, multi-objective lead optimization, database-grounded retrieval

## Abstract

Recently, there has been great interest in AI-based approaches for de novo design of novel drug candidates. However, the generation of useful lead drug candidate compounds requires more than predicting engagement with the desired protein target. Candidate molecules must also be anchored in the real world of medicinal chemistry for their synthesis and modification as well as satisfying multiple drug development-related criteria. Here, we present Nevermore, an AI target-conditioned, database-grounded workflow for prioritizing candidate ligands from large compound libraries. Nevermore uses a geometry-aware protein–ligand affinity oracle to score target-specific binding and perform sparse integer edits in count-based Morgan fingerprint space. Nevermore then retrieves the most structurally similar molecules from public chemical databases. This design enables multi-objective search over predicted affinity and absorption, distribution, metabolism, excretion, and toxicity (ADMET) proxies while keeping all candidates anchored to valid database compounds. We evaluated Nevermore’s performance across three biologically distinct targets: Menin, a protein-interaction target relevant to leukemia; SARS-CoV-2 M^pro^, a viral cysteine protease relevant to antiviral discovery; and epidermal growth factor receptor (EGFR), a kinase-superfamily oncology target with extensive experimentally tested compounds. Nevermore retrieved candidate sets with favorable predicted affinity–property trade-offs. These results support database-grounded fingerprint steering as a practical computational strategy for lead prioritization and for generating testable molecular hypotheses, although the prioritized candidates remain predictions, requiring follow-up experimental validation.

## Simple Summary

Drug discovery requires identifying molecules that bind to a biological target while also satisfying drug-like properties related to safety and developability. Because experimental testing of large compound libraries is expensive and time-consuming, computational workflows can help prioritize promising candidates before laboratory validation. In this study, we present Nevermore, a target-conditioned AI workflow for lead compound prioritization that combines protein–ligand representation learning, multi-objective optimization, and database-grounded molecular retrieval. Rather than generating entirely new molecules, Nevermore performs controlled edits in molecular fingerprint space and retrieves nearby real compounds from large chemical libraries, keeping candidates chemically valid and traceable. As case studies, we selected three potential drug targets: the human proteins menin and EGFR and the SARS-CoV-2 viral protein Mpro. Nevermore was able to identify candidate compound sets with favorable predicted binding–property trade-offs and provides inspectable fingerprint-edit analyses that suggest testable chemical hypotheses for experimental follow-up.

## 1. Introduction

Modern drug discovery relies on computational methods to help prioritize potential drug candidates before costly synthesis and experimental assays. A central component of this process is protein–ligand binding affinity prediction, which estimates how strongly a compound may interact with a therapeutic protein target. Deep learning methods, including structure-aware scoring and complex-prediction models, have improved predictive performance in a range of settings [1,2]. Nevertheless, predicted potency alone is rarely sufficient for translational success. Drug candidates continue to exhibit substantial attrition during development, and late-stage failures are frequently associated with safety liabilities, inadequate efficacy, pharmacokinetic limitations, and toxicity [3,4,5].

These challenges have motivated computational pipelines that jointly consider target binding and developability-related constraints, including ADMET properties, namely, absorption, distribution, metabolism, excretion, and toxicity [6,7]. Recent inverse-QSAR (quantitative structure–activity relationship) and multi-objective optimization frameworks operationalize this goal by searching for molecular representation-level changes that improve predicted efficacy while maintaining drug-likeness and safety profiles [8,9]. In parallel, descriptor- and fingerprint-based approaches have shown that well-chosen molecular representations can remain competitive in data-constrained regimes, especially when coupled with sample-efficient black-box optimization over large compound libraries [10,11]. Taken together, these lines of work motivate hybrid strategies that combine target-conditioned affinity prediction, interpretable molecular representations, and constraint-aware optimization to support computational hit prioritization and lead refinement [12].

**Figure 1:**
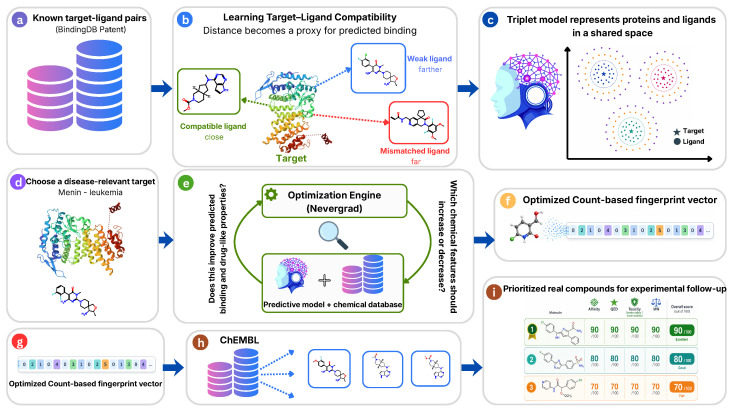
Conceptual workflow of Nevermore for target-conditioned lead prioritization. (**a**–**c**) Known target–ligand pairs are used to learn a shared representation space in which compatible ligands are close to their targets and weak or mismatched ligands are farther away. (**d**–**f**) For a selected disease-relevant target and starting ligand, an optimization engine searches for molecular fingerprint changes that improve the predicted binding–property trade-off, producing an optimized count-based fingerprint vector. (**g**–**i**) The resultant vector is passed to the retrieval stage, where nearby real compounds are retrieved from ChEMBL and ranked for follow-up using predicted affinity and developability-related properties, including QED, hERG risk, molecular weight, and an overall trade-off score.

As illustrated in Figure 1, we present Nevermore, a target-conditioned framework that bridges binding-affinity modeling with multi-objective refinement via feature-space steering and database-anchored retrieval. Rather than generating entirely new molecular structures, Nevermore searches in an editable count-based molecular fingerprint space and retrieves nearby real compounds from a large chemical library. This design keeps the final candidates chemically valid and traceable, while still allowing the model to ask how local chemical-feature patterns should change for a specific target.

The central idea is that target–ligand compatibility can be represented geometrically. Known target–ligand pairs are used to learn a shared space in which compatible ligands are placed closer to their protein targets, whereas weak or mismatched ligands are placed farther away. Given a disease-relevant target and a starting ligand, Nevermore proposes sparse fingerprint edits and evaluates whether the resulting feature profile improves the predicted binding–property trade-off. The optimized fingerprint is then projected back into a compound library such as ChEMBL, and retrieved molecules are ranked using predicted affinity together with developability-related criteria.

We evaluated Nevermore across three diverse and challenging disease-related targets. Menin (MEN1_HUMAN; UniProt: O00255) is a protein–protein interaction regulator whose inhibition has emerged as a clinically relevant strategy in molecularly defined acute leukemias, including KMT2A-rearranged and NPM1-mutant disease, where small molecules such as revumenib disrupt the menin–KMT2A interaction [13]. SARS-CoV-2 main protease, also known as Mpro or 3CL-protease (R1A_SARS2; UniProt: P0DTC1), is a viral cysteine protease required for coronavirus polyprotein processing and has been the focus of structure-enabled inhibitor discovery efforts [14]. Epidermal growth factor receptor (EGFR_HUMAN; UniProt: P00533) is a kinase-superfamily oncology target in which somatic mutations can be linked to therapeutic response, providing a well-established example of target-driven small-molecule intervention [15]. Together, these targets span distinct biological and medicinal-chemistry contexts, a protein–protein interaction regulator, a viral enzyme, and an oncogenic kinase, but they share the same practical question: which real compounds should be prioritized for experimental follow-up?

Across these studies, we show that the Nevermore framework prioritizes candidate sets with favorable predicted affinity–property trade-offs compared with random sampling, baseline-centered fingerprint retrieval, and learned embedding-based retrieval. Because optimization proceeds through sparse feature edits and database-grounded retrieval, Nevermore also allows users to inspect which fingerprint dimensions change, which retrieved molecules support those changes, and which chemical motifs may be associated with improved predicted affinity–property profiles. Technical details of the representation learning model, fingerprint optimization, ADMET proxy scoring, and nearest-neighbor retrieval procedure are provided in the Section 3.

## 2. Related Work

**Protein–ligand affinity prediction and joint embedding models.** Protein–ligand affinity prediction depends critically on how proteins and small molecules are represented, and has driven a progression from end-to-end sequence models to pretrained joint-embedding approaches. Early sequence-based predictors encoded protein sequences and ligand SMILES with convolutional networks and regressed affinity from fused features (e.g., DeepDTA) [16], with subsequent variants refining the convolutional interaction modeling [17]. A parallel line of work represents ligands as molecular graphs and couples graph encoders with protein sequence encoders to learn end-to-end interaction predictors (e.g., GNN-CPI) [18]. More recently, transformer architectures improved cross-modal modeling by applying attention over SMILES and protein sequences; MolTrans is a representative example that learns interaction-aware features from both modalities [19].

A complementary viewpoint treats drug–target interaction (DTI) as representation alignment: proteins and ligands are first embedded with strong pretrained encoders, then lightweight projection heads are trained so that interacting pairs are close in a shared metric space while mismatched pairs are separated. Protein language models, such as ESM [20] and ProtTrans-style models [21], provide transferable protein features; while molecular foundation models, such as MolE [22], and SMILES-pretrained transformers, such as ChemBERTa [23], provide general molecular representations. Contrastive and metric-learning objectives explicitly shape this geometry, often improving robustness by enforcing relative structure (“which pairs should be closer”) rather than relying only on pointwise regression [24,25,26,27]; alternatively inexpensive ligand descriptors, such as circular (ECFP/Morgan) fingerprints, can complement learned embeddings, providing a compact and effective representation for large-library prioritization and retrieval [12,24,25].

**Multi-objective lead optimization.** Lead optimization is inherently multi-objective: candidates must improve affinity while also satisfying development constraints such as solubility, permeability, toxicity risk, and feasibility of synthesis. A common formulation is goal-directed optimization, where a proposal mechanism is coupled to fast surrogate evaluators, often including learned ADMET predictors [7].

One influential family of methods performs optimization in a learned continuous latent space. Gómez-Bombarelli et al. trained an encoder and decoder to map discrete molecular representations into a continuous vector space, together with property predictors defined on that space [28]. This enables molecular search through sampling and local perturbations around known molecules, as well as gradient-based updates in latent space to improve target properties before decoding back to discrete structures [28]. Related de novo directions include reinforcement learning over SMILES strings [29,30], graph-based construction or editing constrained by chemical validity [31], and evolutionary approaches that use fragment-based recombination or genetic operators to iteratively refine candidates under multiple objectives [32]. For example, Zhavoronkov et al. [33] demonstrated generative tensorial reinforcement learning for rapid identification of DDR1 kinase inhibitors.

De novo generative methods can explore chemical space beyond existing compound libraries, which is a major advantage when novel scaffolds are desired. However, this flexibility also introduces challenges, including chemical validity, synthesizability, reward sparsity, and exploitation of imperfect surrogate objectives. In contrast, database-anchored retrieval strategies restrict the search to existing molecules, which can reduce generative freedom but improve the chemical validity, traceability, and practical reviewability of prioritized candidates.

Because optimization can exploit imperfections in surrogate objectives, benchmarking has emphasized validity, diversity, and distribution shift under proxy-driven search. GuacaMol provides standardized goal-directed tasks and highlights common failure modes when optimizing against approximate property or similarity objectives [34]. In target-conditioned pipelines, docking or physics-inspired scoring can add structural signal, but it is typically more expensive than learned or descriptor-based surrogates and is therefore harder to use as a tight inner-loop objective [32].

An alternative direction frames molecular design as black-box optimization over engineered feature spaces. In this setting, descriptors and fingerprints provide explicit, fixed-length representations that can be edited and evaluated efficiently, enabling uncertainty-aware surrogate modeling and sample-efficient Bayesian optimization. Multi-objective variants use Pareto-aware acquisition functions to explore trade-offs directly in objective space, and empirical studies have reported advantages over fixed-weight scalarization baselines in evaluation-limited regimes, including improved Pareto coverage and diversity [35]. Beyond acquisition design, recent work improves practicality by restricting optimization to adaptively selected subspaces over large descriptor libraries, which can improve data efficiency and interpretability [10]. Related directions also couple proposal mechanisms with multi-objective guidance, including LLM-guided frameworks for iterative lead improvement [36]. These feature-space formulations are particularly compatible with target-conditioned pipelines, where affinity is optimized jointly with developability objectives, a setting that we explicitly address in this work.

## 3. Materials and Methods

### 3.1. Method Overview

Nevermore is designed as a target-conditioned computational workflow for prioritizing candidate ligands before experimental testing. Instead of directly generating new molecular structures, the method searches in an editable molecular-feature space and then retrieves nearby real compounds from a reference library. This design keeps the workflow grounded in valid database molecules and makes the optimization process easier to inspect.

**Figure 2:**
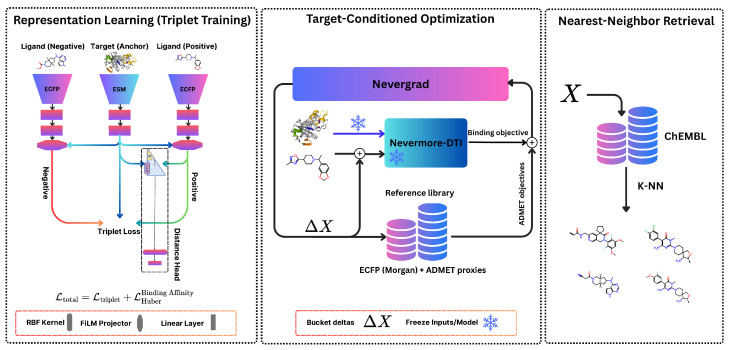
**Nevermore** **pipeline.** (**Left**) **Representation learning.** A geometry-aware DTI oracle is trained with triplet learning to align protein embeddings from ESM and ligand features from count-based extended-connectivity fingerprints (ECFP; Morgan fingerprints) in a shared representation space, using a FiLM projector and a distance-based RBF affinity head. (**Middle**) **Target-conditioned optimization with projection in the loop.** Given a target protein and a baseline ligand fingerprint *X*, Nevergrad [37] proposes sparse integer bucket edits ΔX. The DTI oracle is kept frozen during optimization, while a multi-objective score combines the predicted binding objective with ADMET/property-violation penalties estimated from a reference library. (**Right**) **Nearest-neighbor retrieval.** Each edited fingerprint is projected back to valid molecules by *K*-NN search in a large compound library, such as ChEMBL, yielding database-matched candidate ligands for downstream review.

Figure 2 summarizes the technical workflow. The method has three main stages. First, a protein–ligand affinity oracle is trained to score whether a ligand is compatible with a specific protein target. Proteins are represented using pretrained protein embeddings, and ligands are represented using count-based Morgan fingerprints. The model learns a shared target–ligand representation space in which compatible pairs are encouraged to be closer than weak or mismatched pairs.

Second, after the affinity oracle is trained, its weights are frozen and used as a fixed scoring function during optimization. Given a target protein and a starting ligand, Nevermore performs sparse edits to selected coordinates of the ligand fingerprint. These edits are not interpreted as direct atom-by-atom chemical transformations. Instead, they represent changes in local chemical-feature patterns that may improve the predicted binding–property trade-off for the selected target.

Third, because an edited fingerprint may not correspond to a valid molecule, each optimized fingerprint is projected back into a real compound library using nearest-neighbor retrieval. In this work, we use a ChEMBL-derived library with precomputed Morgan fingerprints and property annotations. The retrieved molecules are then evaluated using predicted affinity together with ADMET/property-related criteria, including molecular weight, QED, logP, hERG liability, and related constraint penalties. The final candidates are ranked in a Pareto-aware manner, emphasizing trade-offs rather than affinity alone.

Thus, Nevermore should be interpreted as a computational lead-prioritization and hypothesis-generation workflow. The reported candidates are valid retrieved molecules, and their affinity and ADMET values are model-based predictions rather than experimental measurements. The fingerprint edits provide traceable chemical-feature hypotheses, but they do not prove a mechanistic binding effect. Detailed architecture, optimization, ADMET scoring, retrieval, and implementation settings are described in the following subsections and in Appendix A.

### 3.2. Affinity Oracle

#### 3.2.1. Architecture

As the scoring oracle in Nevermore, we use a geometry-aware DTI model following Firm–DTI [25]. Proteins are encoded with a frozen ESM2 protein language model (mean-pooled sequence embedding), and ligands are encoded as RDKit count-based Morgan fingerprints (radius r=3, B=1024 bins; sensitivity analysis is reported in the Appendix B). Each modality is passed through a learned projection MLP into a shared *d*-dimensional latent space. We denote the projected protein (target) embedding by $z_{t}\in R^{d}$ and the projected ligand (drug) embedding by $z_{d}\in R^{d}$.

To model target-conditioned interactions, we use a Feature-wise Linear Modulation (FiLM) layer [38], which conditions one representation using another through feature-wise affine transformations. Here, the protein embedding generates element-wise scaling and shifting coefficients that modulate the ligand embedding, enabling the model to adapt ligand features according to the target context. Formally, we FiLM-condition the ligand embedding on the protein embedding:

$${\tilde{z}}_{d}\;=\;\mathrm{FiLM}\left(z_{d}|z_{t}\right)\;=\;\gamma \left(z_{t}\right)⊙z_{d}+\beta \left(z_{t}\right)$$

where $\gamma \left(\cdot \right),\beta \left(\cdot \right):R^{d}\to R^{d}$ are learned linear maps that produce element-wise scaling and shifting vectors, and ⊙ denotes element-wise multiplication. We $\ell_{2}$-normalize the resulting embeddings and compute a cosine distance:

$$\mathrm{dist}\left({\tilde{z}}_{d},z_{t}\right)=1-\frac{{\tilde{z}}_{d}\cdot z_{t}}{∥{\tilde{z}}_{d}∥\;∥z_{t}∥}.$$

Finally, affinity is predicted via an RBF expansion over this distance with *k* centers ${\left\{\mu_{j}\right\}}_{j=1}^{k}$ (evenly spaced in [0,2]):

$$\phi_{j}=\exp\;\left(-\frac{{\left(\mathrm{dist}\left({\tilde{z}}_{d},z_{t}\right)-\mu_{j}\right)}^{2}}{2\sigma^{2}}\right),\;j=1,\ldots ,k,\;\hat{y}=W\phi +b,$$

where $\phi =[\phi_{1},\ldots ,\phi_{k}]$ and $\hat{y}$ is the predicted log-affinity.

#### 3.2.2. Datasets

The oracle is trained on Therapeutics Data Commons (TDC) DTI-DG drug–target pairs [39], using experimentally reported affinity labels (e.g., $pK_{d}$, $pIC_{50}$, or $pXC_{50}$). For triplet construction, positives are chosen as higher-affinity compounds for a given protein, and negatives as lower-affinity or cross-target compounds.

#### 3.2.3. Training

We train the oracle once, offline, with AdamW, using the combined objective

$$L=L_{\mathrm{triplet}}+L_{\mathrm{Huber}}$$

where $L_{\mathrm{triplet}}$ enforces a margin between positive and negative protein–ligand pairs in the embedding space and $L_{\mathrm{Huber}}$ regresses $\hat{y}$ onto the experimental log-affinity labels. Full details of the training protocol, hyperparameter settings, dataset split, and model-selection procedure are provided in Appendix C. The best checkpoint is selected by validation correlation and then frozen before any molecular optimization is performed.

Freezing the oracle is a deliberate design choice. It separates affinity-model training from candidate search, so all optimization runs are scored by the same fixed function and can be compared under identical conditions. This also prevents the model from changing in response to the candidates proposed during optimization, making the resulting rankings easier to reproduce and interpret. In practice, Nevermore therefore uses the trained model as a black-box scoring oracle: the optimizer can query predicted affinity values, but it cannot update the model weights. The limitation of this design is that any calibration error, target-domain bias, or uncertainty in the frozen oracle is inherited by the downstream candidate ranking. For this reason, the reported affinity values should be interpreted as predicted prioritization scores rather than experimental binding measurements.

### 3.3. ADMET and Property Screening

To characterize the drug-likeness and safety-related properties of each ligand, we assemble a panel of in silico property annotations for every molecule in the training, validation, and retrieval pools. These annotations combine structure-computable descriptors and rule-based filters with a small number of learned biological proxy endpoints. Specifically, molecular weight (MW), logP, quantitative estimate of drug-likeness (QED), and Lipinski-style summary features are computed deterministically from the molecular graph using standard cheminformatics routines, whereas endpoints such as human ether-à-go-go-related gene (hERG) blockade probability and human intestinal absorption (HIA_Hou), which are not directly computable from SMILES alone, are obtained from ADMET-AI [7]. Thus, the property annotations are either directly recomputable from the molecular structure or obtained from a cited, publicly described ADMET prediction framework. We run ADMET-AI once, offline, for the relevant molecules and store the resulting annotations in a tabular database, keyed by a unique molecule identifier.

In this work, structure-derived descriptors provide the main optimization constraints, while learned ADMET endpoints are used more conservatively as auxiliary screening proxies. This distinction is important because descriptors such as molecular weight, logP, QED, and Lipinski-style summaries are deterministic, whereas hERG and HIA predictions may inherit calibration error or chemical-domain bias from the underlying predictive models. Accordingly, Nevermore does not interpret these predicted endpoints as experimentally validated safety or pharmacokinetic outcomes; instead, they serve as inexpensive computational filters for prioritizing molecules within the retrieval pool. The resulting property table provides, for each molecule, a fixed-length vector of annotations that can be queried during optimization and retrieval, and later summarized in the case studies to compare baseline ligands and retrieved candidates.

### 3.4. Nevergrad Feature-Space Optimization

We use Nevergrad [37] as a derivative-free optimizer to search over an editable fingerprint space rather than directly modifying molecular graphs. This choice is convenient in our setting because the search variable is discrete (count buckets), the projection step maps edits back to real library molecules, and the resulting pipeline is effectively non-differentiable. We parameterize this space with count-based ECFP (Morgan) fingerprints, which capture local chemical environments in a fixed-length vector amenable to discrete steering and are a widely used, competitive baseline in molecular property modeling compared to coarser structural keys (e.g., MACCS) or more expensive alternatives [10]. While hashed fingerprints can be coarse and non-invertible due to collisions, Nevermore avoids explicit decoding by grounding edits through database retrieval.

Given a target protein sequence *t* and a candidate ligand *s*, our frozen DTI oracle returns a predicted binding affinity $\hat{y}\left(t,s\right)$. In addition, we associate each library compound with precomputed auxiliary quantities (e.g., its Morgan count fingerprint and developability proxies such as ADMET endpoints). During optimization, once Nevergrad [37] proposes a steered fingerprint, mapping it to candidate molecules and evaluating constraint penalties can be implemented efficiently using nearest-neighbor retrieval followed by lookup of the stored properties for the retrieved compounds.

#### 3.4.1. Decision Variables and Edited Fingerprint

Let $X\left(s\right)\in N^{B}$ denote the count-based Morgan fingerprint of ligand *s* with *B* buckets. Starting from a baseline ligand *s*, we select a small editable set of buckets $S=\left\{S_{1},\ldots ,S_{m}\right\}\subset \left\{1,\ldots ,B\right\}$, with m≪B. Nevergrad proposes a real-valued edit vector $\delta \in R^{m}$, which we discretize as

$$\Delta =\mathrm{round}\left(\delta \right)\in Z^{m}.$$

The edited fingerprint is then

(1) $$\tilde{X}\left(s,\delta \right)=\max\;\left(X\left(s\right)+M_{S}\Delta ,\;0\right),$$

where $M_{S}\in {\left\{0,1\right\}}^{B\times m}$ is the insertion matrix that places the *m* editable coordinates into the full *B*-dimensional fingerprint, and the max(·,0) operation is applied element-wise to enforce non-negative bucket counts.

#### 3.4.2. Projection in the Loop (Top-*K* Neighbor Evaluation)

Because $\tilde{X}\left(s,\delta \right)$ may not correspond to a valid molecule, we project each proposal onto real ligands in a reference library D by retrieving the *K* nearest neighbors under a weighted $\ell_{1}$ distance:

(2) $$N_{K}\left(s,\delta \right)={\mathrm{KNN}}_{s^{\prime }\in D}\left(\tilde{X}\left(s,\delta \right);\;d_{c}\left(\cdot ,\cdot ;\Delta \right)\right),$$

where KNN returns the *K* compounds with smallest distance, and 

(3) $$d_{c}\left(x,y;\Delta \right)=\sum_{k=1}^{B}c_{k}\left(\Delta \right)\;\left|x_{k}-y_{k}\right|.$$

$$c_{k}\left(\Delta \right)=\left\{\begin{array}{cc} 1+\lambda |\Delta_{r}|, & \mathrm{if}\;k=S_{r}\;\mathrm{for}\;\mathrm{some}\;r\in \left\{1,\ldots ,m\right\},\\ 1, & \mathrm{otherwise}, \end{array}\right.$$

so that buckets explicitly modified by Nevergrad are emphasized during retrieval.

The neighborhood size *K* controls how broadly each edited fingerprint is projected into the real compound library. Smaller *K* values restrict evaluation to the closest database matches, whereas larger *K* values average the objective over a broader set of feasible retrieved molecules. Importantly, edited fingerprints are used only as search variables; affinity and property scores in the optimization loop are evaluated after projection to real library compounds.

#### 3.4.3. Joint Objective with Affinity and ADMET Penalties

For each retrieved neighbor $s^{\prime }\in N_{K}\left(s,\delta \right)$, we evaluate predicted affinity and an ADMET penalty using precomputed library endpoints. Let $a_{j}\left(s^{\prime }\right)$ denote ADMET endpoint j∈A for compound $s^{\prime }$, with acceptable range $\left[\ell_{j},u_{j}\right]$ and penalty weight $\lambda_{j}$. We define

(4) $$L\left(t,s^{\prime }\right)=-\hat{y}\left(t,s^{\prime }\right)+\beta \sum_{j\in A}\lambda_{j}\left({\left[\ell_{j}-a_{j}\left(s^{\prime }\right)\right]}_{+}^{2}+{\left[a_{j}\left(s^{\prime }\right)-u_{j}\right]}_{+}^{2}\right),$$

where ${\left[x\right]}_{+}=\max\left(x,0\right)$ and β controls the trade-off between affinity and ADMET feasibility. The Nevergrad objective is the mean loss over the retrieved set:

(5) $$\min_{\delta }\frac{1}{K}\sum_{s^{\prime }\in N_{K}\left(s,\delta \right)}L\left(t,s^{\prime }\right).$$

#### 3.4.4. Retrieval Library

For projection and attribute lookup, we use a ChEMBL-derived retrieval set containing approximately 2.9 million compounds [40]. Count-based Morgan fingerprints and ADMET/property annotations are precomputed once offline, allowing edited fingerprints to be projected efficiently to database molecules and enabling rapid lookup of property values during optimization.

## 4. Results

### 4.1. DTI Oracle Model for Binding-Affinity Optimization and Interaction Prediction

#### 4.1.1. Binding-Affinity Oracle (Regression)

**Figure 3:**
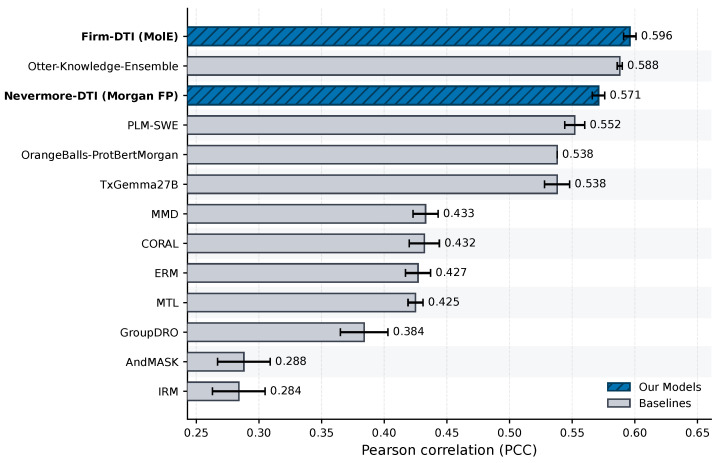
BindingDB Patent (TDC) affinity prediction. Ranked comparison by Pearson correlation (PCC) between predicted and experimental binding affinity. Firm–DTI (MolE) achieves the highest PCC, followed by Otter-Knowledge-Ensemble and Nevermore–DTI (Morgan FP).

We train a geometry-aware protein–ligand affinity predictor to serve as the scoring oracle in Nevermore. We evaluate on the BindingDB Patent benchmark (TDC) [39], using the Pearson correlation coefficient (PCC) between predicted and experimental affinities. The oracle encodes the target protein with a frozen protein language model (PLM) and represents ligands using a count-based Morgan fingerprint (ECFP-style), which is then projected into a shared latent space. Figure 3 shows that our Morgan-fingerprint oracle achieves a PCC of 0.571, outperforming several previously reported baselines, including PLM-SWE (0.552) [26] and TxGemma27B (0.538) [41], while remaining close to Otter-Knowledge-Ensemble (0.588) [42]. Additional baseline results shown in the figure are taken from Huang et al. [39]. Among the variants we tested, the MolE-based ligand encoder achieves the highest PCC (0.596), whereas the Morgan-fingerprint variant is approximately 2.5% lower. We nevertheless use the Morgan-fingerprint oracle in Nevermore because it provides a directly editable discrete feature space (fingerprint buckets), enabling efficient Nevergrad search and straightforward projection back to valid library molecules.

#### 4.1.2. DTI Classification (Binary Interaction)

In addition to affinity regression, we train the same geometry-aware protein–ligand model as a binary drug–target interaction (DTI) classifier by replacing the regression head with a sigmoid classification head and optimizing cross-entropy loss [25]. We evaluate on three standard benchmarks, DAVIS [43], BindingDB [44], and BIOSNAP (ChG-Miner) [45], reporting AUROC and AUPR. All DTI classification experiments were repeated across five random seeds, and the results are reported as mean ± s.e.m. across these runs. Statistical robustness was further assessed using an approximate Kruskal–Wallis sensitivity analysis, where pseudo-seed values were generated from Gaussian distributions parameterized by the reported mean and estimated standard deviation across 2000 Monte Carlo reconstructions. Following MolTrans [19], we binarize the DAVIS and BindingDB datasets using a dissociation constant threshold of 30nM, where interactions with $K_{d}<30\;\mathrm{nM}$ are labeled positive and those with $K_{d}\geq 30\;\mathrm{nM}$ are labeled negative.

Table 1 summarizes the results for two ligand representations within this architecture: a MolE-based ligand encoder and an editable count-based Morgan fingerprint (Morgan FP). Across the datasets, the MolE variant yields the strongest overall performance, while the Morgan-FP variant remains competitive and typically ranks second, outperforming or matching several established baselines. The approximate Kruskal–Wallis analysis indicated statistically robust differences for most benchmark–metric pairs, except BindingDB AUROC.

### 4.2. Sensitivity to Objective Weight, Retrieval Metric, and Edit Budget

Nevermore optimizes sparse edits to Morgan count buckets with Nevergrad, while staying database-grounded via projection in the loop: after each edit, we retrieve the top-*K* nearest library molecules and summarize the retrieved set by predicted affinity and precomputed ADMET endpoints. The default scalarization uses β=1, corresponding to equal weighting of the normalized affinity and property-penalty terms; alternative objective weights are evaluated in the sensitivity analysis below. We report (i) ΔAffinity (vs. the baseline ligand), (ii) Mol_Aff (mean affinity over the retrieved top-*K*), and (iii) mean ADMET endpoints over the same set (MW, logP, hERG risk, QED; sign-flipped where needed so higher is better). For comparison across metrics, we z-normalize columns across runs and visualize sweep heatmaps, with stars marking the best configuration(s) per metric. We evaluate 10 protein targets held out from our training, and for stable statistics we summarize K=100 retrieved molecules per target (1000 total).

#### Hyperparameter Sweeps and Trade-Offs

**Figure 4:**
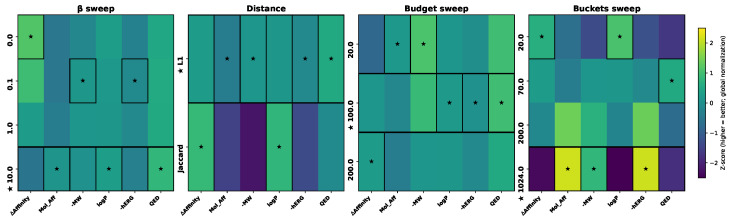
Optimization dynamics across objectives and search settings. Each panel varies one factor (left to right: β, distance, budget, editable buckets). Columns show z-normalized metrics (higher is better): ΔAffinity, Mol_Aff, −MW, logP, −hERG, QED. Stars mark best configuration(s) per column within each panel.

Figure 4 summarizes four controlled sweeps: (i) multi-objective weight *β*, (ii) retrieval distance (Jaccard vs. weighted $\ell_{1}$), (iii) optimization budget (Nevergrad iterations), and (iv) number of editable buckets.

Increasing *β* shifts the search from affinity-only toward constraint-aware optimization, revealing a consistent trade-off: low-*β* settings tend to maximize ΔAffinity, whereas higher *β* improves the projected neighborhood (higher Mol_Aff and QED) and reduces liability-aligned endpoints such as MW and hERG risk (after sign-flipping). Across distances, weighted $\ell_{1}$ retrieval produces more stable and higher-quality neighborhoods than Jaccard, consistent with magnitude-aware matching being better aligned with Morgan-count edits. Increasing the iteration budget generally improves ΔAffinity, but retrieved-set ADMET/QED values often peak at moderate budgets, suggesting diminishing returns once the optimizer begins exploiting feature edits that project less reliably. Finally, increasing the editable bucket set |S| gives the optimizer more flexibility, but very large bucket sets can make the optimized fingerprint harder to match to real compounds in the database. Small |S| values can produce strong ΔAffinity improvements, whereas intermediate or larger |S| values often improve projected-neighborhood properties such as Mol_Aff, hERG, MW, or QED. Thus, no single bucket size is optimal for all objectives.

Overall, the sweeps show that improvements in the optimized fingerprint space do not always translate directly to better database-retrieved compounds. Nevergrad may suggest fingerprint edits that improve predicted ΔAffinity, but these edits may not closely correspond to real compounds in the library. Because the nearest-neighbor projection step can change the retrieved molecules abruptly, the settings that maximize ΔAffinity do not necessarily give the best Mol_Aff or ADMET trends after projection.

Based on these controlled sweeps, we used 70 editable buckets for the subsequent menin, SARS-CoV-2 M^pro^, and EGFR case studies; weighted $\ell_{1}$ projection, retrieval neighborhood size K=10, and β=1 in Equation (4). This configuration was selected as a practical trade-off: it provides enough edit capacity to modify multiple property-related signals while avoiding the less stable projected neighborhoods observed when the edited fingerprint moves too far from well-supported regions of the retrieval library. The value β=1 was used as a balanced setting between predicted affinity and property-violation penalties, rather than as an affinity-only or target-specific tuned regime. Additional sensitivity analysis for the retrieval neighborhood size *K* is provided in Appendix G.

### 4.3. Case Study: Cross-Target Evaluation of Multi-Objective Ligand Prioritization

#### 4.3.1. Target Selection and Baseline Ligands

We selected two biologically distinct targets that present different ligand-optimization settings. Menin is an oncology-relevant target, where small-molecule inhibitors are designed to disrupt menin–protein interactions rather than inhibit an intrinsic enzymatic activity [46,47]. This provides a challenging protein–protein interaction (PPI)-like binding context. In contrast, SARS-CoV-2 M^pro^ is a viral cysteine protease required for coronavirus polyprotein processing, and inhibitors target its catalytic substrate-binding cleft [48]. Together, these two targets provide complementary case studies: one centered on a protein–protein interaction interface and one on an enzyme active site.

As a stringent starting point for the menin case study, we use revumenib (Revuforj) [49] as the baseline ligand. Revumenib is an FDA-approved menin inhibitor used in molecularly defined acute leukemias. Because this scaffold has already undergone extensive therapeutic optimization, the menin case provides a demanding test of whether the proposed workflow can identify candidate sets with improved predicted affinity–property trade-offs relative to local similarity-based retrieval.

For M^pro^, we use cinanserin [50] as the baseline ligand. Cinanserin provides a chemically plausible starting scaffold for antiviral-relevant prioritization, while its reported acute oral toxicity warning motivates evaluation beyond predicted affinity alone. This makes the M^pro^ case useful for testing whether multi-objective prioritization can balance predicted binding with property-related constraints.

For each target, we keep the baseline ligand fixed and generate a fixed budget of candidates (*N* = 100) from the same ChEMBL-scale library (∼2.9 M compounds) using four strategies: (i) Random sampling from the library; (ii) RDKFP+Tversky similarity retrieval, where RDKit topological fingerprints are computed for all library molecules and the nearest neighbors to the baseline are retrieved under the Tversky similarity, an asymmetric generalization of Jaccard/Tanimoto similarity that can emphasize containment of baseline substructures; (iii) MolE embedding retrieval, where the baseline ligand and all library molecules are encoded using the MolE molecular embedding model [22], and nearest neighbors are retrieved in the learned embedding space; and (iv) Nevermore, which performs multi-objective optimization in fingerprint count-space, proposes discrete bucket edits, and then projects each proposal back to valid molecules via nearest-neighbor retrieval from the same library. Nevermore uses 70 editable fingerprint buckets, weighted $\ell_{1}$ projection, and β=1 in the multi-objective loss.

Across both targets, Nevermore provides the most favorable predicted affinity–property trade-off among the evaluated candidate-generation strategies. On SARS-CoV-2 M^pro^, Nevermore achieves the highest mean predicted affinity among the generated candidate sets (7.283), while also reducing the predicted hERG liability score relative to RDKFP+Tversky (0.411 vs. 0.500) and MolE retrieval (0.411 vs. 0.933). It also improves QED relative to both RDKFP+Tversky and MolE (0.740 vs. 0.676 and 0.668, respectively), while maintaining the lowest mean molecular weight among the generated candidates. On menin, the improvements are more pronounced: Nevermore achieves higher predicted affinity than RDKFP+Tversky and MolE (7.060 vs. 6.934 and 6.429), while also producing lower predicted hERG liability, higher QED, and lower molecular weight. These results suggest that database-grounded fingerprint steering can prioritize candidate sets with improved predicted binding–property balance compared with random sampling, local fingerprint-similarity retrieval, and embedding-based molecular retrieval.

#### 4.3.2. Interpreting Optimized Bucket Edits: From Fingerprint Changes to Target-Specific Chemical Hypotheses

To relate Nevermore’s optimized feature-space changes to chemical structure, we analyze the edited fingerprint buckets proposed during optimization and map them to representative fragments observed among the retrieved molecules. This analysis is intended to provide a motif-level interpretation of the search process. Rather than treating the optimizer as a black box, we ask which local chemical environments are repeatedly favored or avoided, whether these preferences are shared across objectives, and whether they differ between targets.

A bucket edit can be interpreted as a directional preference in chemical space. Increasing a bucket count biases retrieval toward molecules containing related local substructures, whereas decreasing a bucket count biases retrieval away from them. Because the final candidates are obtained through nearest-neighbor retrieval, these edits do not prescribe exact atom-by-atom modifications. Instead, they indicate which types of chemical environments help move the search toward more favorable predicted affinity–property trade-offs.

**Figure 5:**
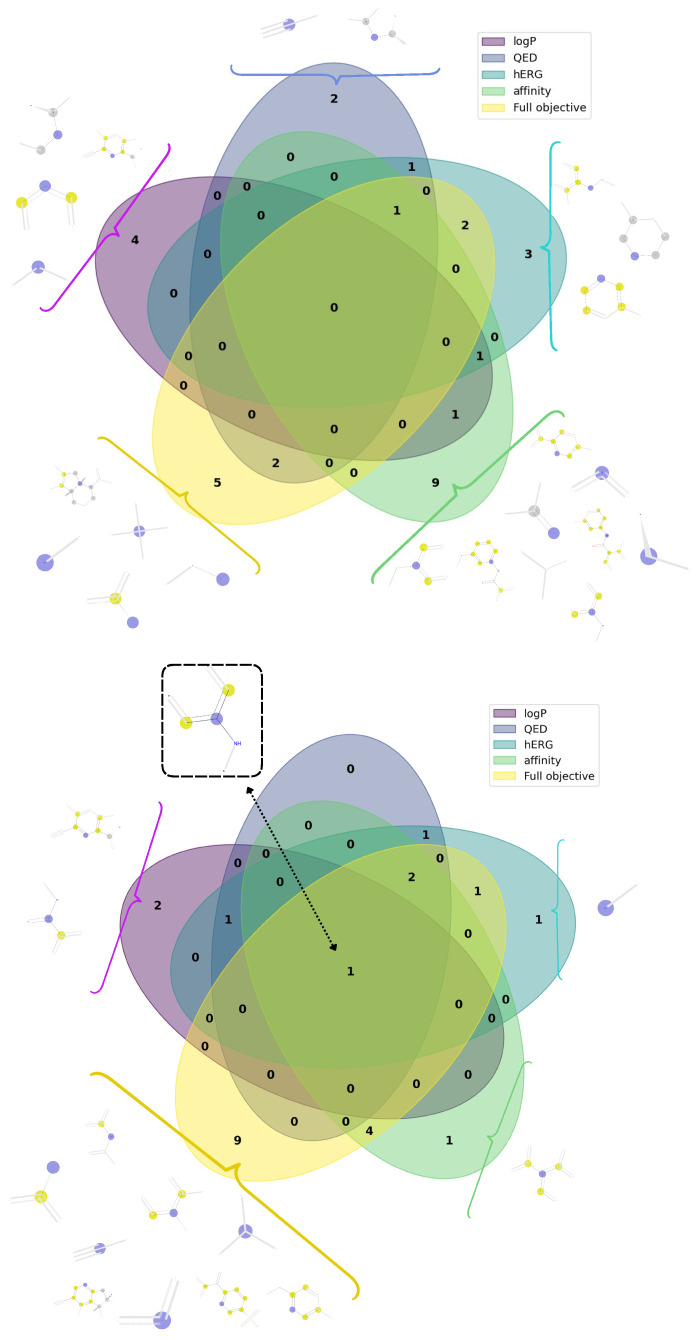
Overlap of optimized bucket edits across objectives. (**Top**): SARS-CoV-2 M^pro^. (**Bottom**): Menin. For each target, we show the sets of Morgan-count buckets whose values were adjusted relative to the baseline ligand (cinanserin for M^pro^, revumenib for menin) during objective-specific optimization (affinity or a single ADMET proxy) and during full multi-objective optimization. Intersections highlight shared edit directions that improve multiple objectives simultaneously, whereas non-overlapping regions identify objective-specific edits that are activated only when a particular proxy constraint is enforced.

We compare the edited-bucket sets obtained when optimizing each objective in isolation, such as affinity-only or single-property-proxy optimization, against the set obtained under the full multi-objective score (Figure 5). Intersections identify recurring bucket-level changes selected across multiple objectives and may indicate motif-level hypotheses compatible with both predicted target engagement and property constraints. Non-overlapping regions identify objective-specific levers, such as edits introduced mainly to reduce a property-proxy penalty. This comparison helps explain why optimizing predicted affinity alone may not produce candidates with favorable developability profiles, and why additional chemical changes are needed when ADMET/property constraints are considered.

To connect these edits to interpretable chemistry, we map edited buckets to representative Morgan bits and extract the corresponding atom environments from retrieved molecules. The resulting fragments provide target-specific chemical hypotheses about the motifs Nevermore tends to select. From a biological and medicinal perspective, these motifs are useful because they suggest which local chemical environments may support binding-site compatibility, alter polarity or hydrophobicity, or improve predicted drug-likeness while maintaining target-conditioned affinity. Since Morgan fingerprints are hashed and a single bucket can correspond to more than one local environment, these fragment assignments should be interpreted as hypothesis-generating chemical associations rather than definitive mechanistic evidence.

For menin (baseline revumenib), Figure 5 shows substantial overlap between the objective-specific edit sets and the full multi-objective run, indicating that several edit directions are broadly beneficial rather than proxy-specific. Notably, the  amide-associated environment emerges as a shared lever across objectives (common to all runs), consistent with carbonyl-containing motifs being associated with improved predicted scores without strongly increasing property-constraint violations. Beyond this shared amide-driven core, the full objective introduces additional edits that map to compact polarity and tuning fragments, including nitrile-like and sulfonyl-like environments, together with halide-associated environments. This pattern is consistent with a “balance” strategy where amide, nitrile, and sulfonyl fragments act as polarity- and property-control motifs, whereas halide-associated edits may help preserve predicted affinity by improving hydrophobic pocket complementarity.

For M^pro^ (baseline cinanserin), the decoded bucket edits reveal chemically intuitive, objective-specific patterns. hERG-driven optimization is associated with neutral polar linker motifs, especially ether-like environments, whereas logP- and QED-oriented runs preferentially introduce compact polar substructures, including nitrile, amide, and ether motifs, that can shift overall polarity without large scaffold changes. In contrast, affinity-driven runs emphasize carbonyl hydrogen-bond-acceptor environments and scaffold/topology edits that may modulate pocket complementarity. The full multi-objective run integrates these pressures by retaining polarity-increasing edits that reduce property-proxy violations while adding hydrophobic tuning through halide-associated environments to preserve or recover predicted binding signal. Together, these edits suggest a target-specific route for balancing predicted affinity with property-related constraints relative to the cinanserin baseline.

#### 4.3.3. Pareto-Style Trade-Offs: Affinity vs. Developability-Related Proxies

**Figure 6:**
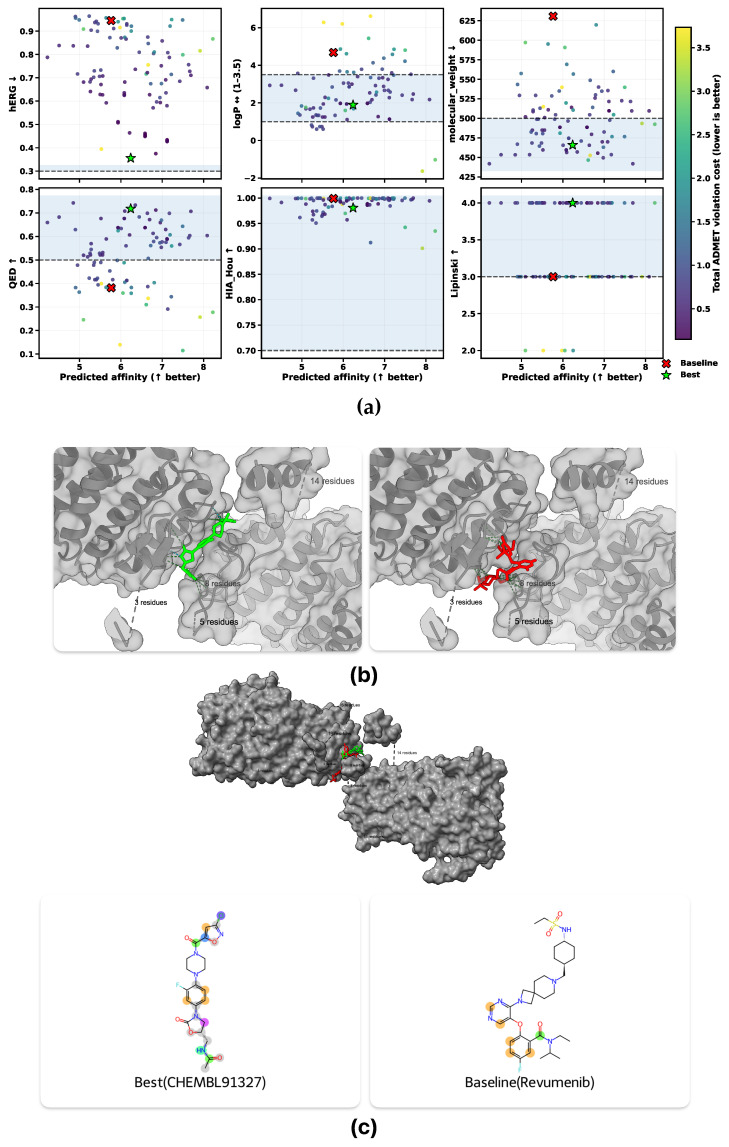
Menin. (**a**) Pareto trade–offs between predicted affinity and ADMET objectives (color: total ADMET–violation cost); baseline revumenib (red ×) vs. selected optimized CHEMBL91327 (green ⋆). (**b**) Pose overlay (baseline red, optimized green) with polar contacts. (**c**) Projection of adjusted Morgan-count buckets onto structure (colored centers, gray environments): baseline realizes **2** adjusted buckets vs. optimized **7**.

**Figure 7:**
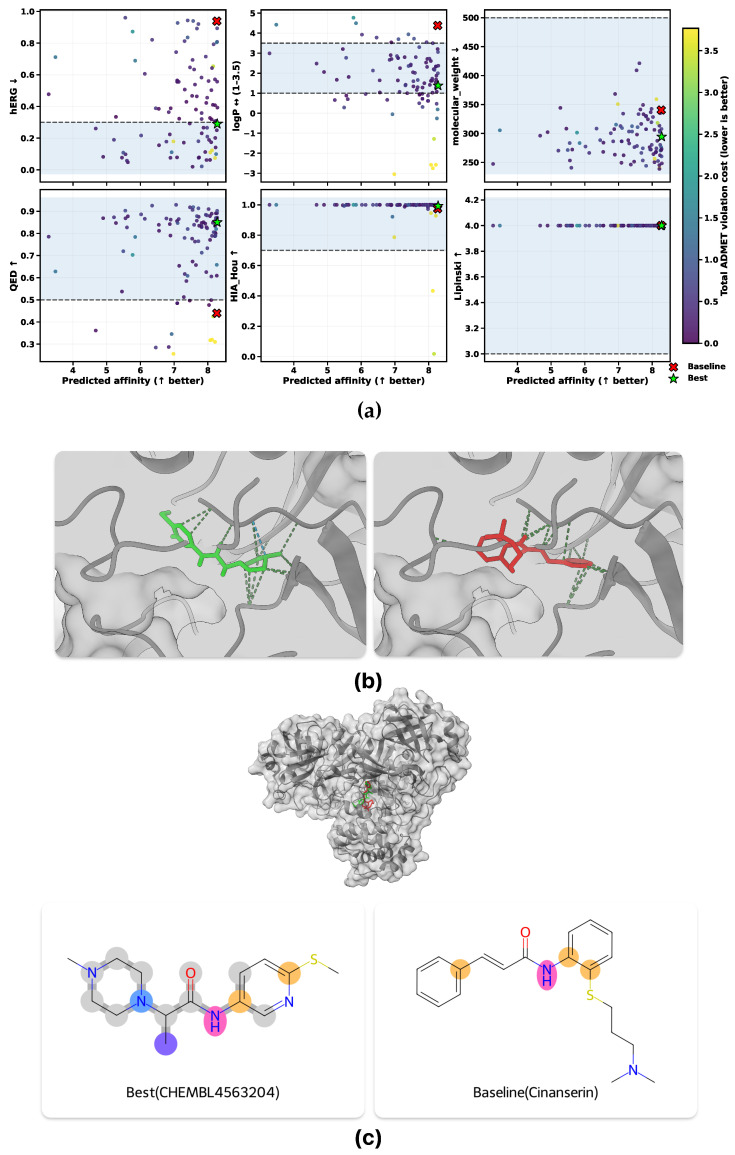
M_pro_. (**a**) Pareto trade–offs between predicted affinity and ADMET objectives (color: total ADMET–violation cost); baseline cinanserin (red ×) vs. selected optimized CHEMBL4563204 (green ⋆). (**b**) Pose overlay (baseline red, optimized green) with polar contacts. (**c**) Adjusted-bucket projection (colored centers, gray environments): baseline realizes **2** adjusted buckets vs. optimized **5**.

Multi-objective lead prioritization is naturally a Pareto-style problem [35], where improving one objective, such as predicted affinity, can come at the expense of other developability-related properties. Therefore, no single metric uniquely defines the preferred candidate. We visualize the retrieved candidate sets using affinity–property trade-off plots (Figure 6 and Figure 7) to show how Nevermore balances predicted binding against multiple property constraints.

In each panel, the x-axis is predicted affinity, where higher values are preferred, and the y-axis is one proxy property: hERG, QED, MW, logP, HIA_Hou, or Lipinski. Dashed lines and shaded regions indicate the preferred direction or acceptable range for each proxy, such as lower hERG and MW, higher QED, and logP, within a target interval. Each point represents one of the N=100 retrieved candidates, and color denotes the total constraint-violation cost across the full proxy panel. The baseline ligand is marked with a red ×. The selected representative candidate, marked with a green ⋆, is chosen as a high-affinity candidate with low total violation cost but it is not necessarily the maximum-affinity molecule.

Menin.

Figure 6 shows a strong predicted multi-objective shift. The baseline ligand violates several proxy constraints, including hERG, MW, and logP, whereas the optimized candidate set moves toward regions with higher predicted affinity and lower total violation cost. The selected representative candidate falls within the preferred ranges for most proxies while also improving predicted affinity. These results suggest that Nevermore can prioritize favorable affinity–property trade-offs even for a challenging PPI-like menin-binding context.

M^pro^.

Figure 7 shows that the cinanserin baseline already lies in a high-predicted-affinity region but violates several property proxies, including elevated hERG, sub-threshold QED, and out-of-range logP. Nevermore shifts the retrieved candidates toward preferred regions across multiple panels: several candidates retain near-baseline predicted affinity while reducing total constraint-violation cost, and the selected representative candidate shows an improved predicted property profile with limited predicted-affinity compromise. This pattern is consistent with the aggregate improvements reported in Table 2.

#### 4.3.4. Bucket-Level Attribution of Discrete Nevermore Edits

After selecting a representative optimized ligand for each target from the Pareto-style trade-off analysis, we examine which fingerprint dimensions were explicitly edited by Nevermore and where the corresponding features appear on the chemical graph. Nevermore operates in Morgan-count space with fixed bucketization: each Morgan feature identifier fid is mapped to a bucket index b=fidmodB, and the optimizer proposes a sparse adjustment vector Δc over buckets. We focus on the adjusted-bucket set

$$A=\{b:\Delta c_{b}\neq 0\},$$

and map these buckets back to molecular structure using RDKit Morgan bitInfo. For each adjusted bucket realized in a molecule, we extract the corresponding Morgan feature center atom and its radius-defined local environment. In the visualization, colored circles mark center atoms associated with adjusted buckets, while gray highlights show the surrounding atoms and bonds defining the corresponding Morgan environments. Quantitatively, we report how many adjusted buckets are realized in each molecule and how many distinct centers and local environments they induce.

Menin (baseline: revumenib; optimized: CHEMBL91327).

The baseline ligand realizes **two** adjusted buckets, whereas the optimized ligand realizes **seven** adjusted buckets. This difference is also reflected in the number of activated feature centers: **six** in the baseline compared with **eleven** in the optimized ligand. The optimized ligand also shows broader structural context supporting these activations, with twenty-nine environment atoms and eighteen bonds highlighted, compared with six environment atoms and no bonds in the baseline. Thus, the optimized candidate exhibits higher coverage of the adjusted-bucket set A and a richer set of bucket-aligned local environments. We interpret this as a traceable structural explanation of how the retrieved molecule reflects the optimizer’s fingerprint edits, rather than as proof that the highlighted atoms directly cause the predicted improvement.

M^pro^ (baseline: cinanserin; optimized: CHEMBL4563204).

The baseline ligand realizes only **two** adjusted buckets, visible as **four** colored center atoms, whereas the optimized ligand realizes **five** adjusted buckets, visible as **six** colored centers. The optimized ligand also induces broader local contexts, with 20 environment atoms and 15 bonds highlighted. This indicates that the optimized molecule matches a larger fraction of the discrete edits proposed in bucket space and represents those edits through more extended local substructures. Adjusted buckets appearing in both ligands correspond to shared local motifs, whereas buckets appearing only in the optimized ligand suggest newly introduced or substantially modified motifs relative to the baseline scaffold. As with the menin case, these mappings provide bucket-level attribution and chemical hypotheses, not definitive mechanistic evidence.

#### 4.3.5. Structural Intuition: Baseline vs. Optimized Ligand Binding Poses

To provide qualitative structural context for the candidates selected by Nevermore, we visualized docking poses for each target using the baseline ligand and the Pareto-selected Nevermore candidate. These docking calculations were used only for supportive visualization and were not used to select, optimize, or re-rank Nevermore candidates.

Briefly, receptors were prepared with AutoDockTools/MGLTools with hydrogen checking, and ligand structures were generated from SMILES using RDKit with added hydrogens, ETKDGv3 conformer embedding, and force-field minimization before conversion to PDBQT format [51,52]. Docking was performed with AutoDock4 Vina using the same receptor preparation, docking-box definition, docking settings, and pose-filtering criteria for the baseline and optimized ligand within each target [53]. The full docking-box coordinates and run parameters are provided in Appendix I.

For each receptor, we retained a representative pose that was clash-free under ChimeraX clash detection and occupied the expected binding-site region [54]. Contact counts and hydrogen-bond displays were computed using identical ChimeraX settings for the baseline and optimized ligand within each target. Because these results can depend on receptor preparation, ligand protonation state, docking-box placement, and pose selection, we interpret the overlays only as qualitative structural illustrations of plausible binding-site accommodation, not as validation of improved binding or biological activity.

Menin (baseline: revumenib; optimized: CHEMBL91327).

For menin, the optimized ligand remains in the same functional binding region as the baseline while showing greater contact density under the selected pose and contact definition. The optimized pose yields more protein–ligand contacts than the baseline pose (baseline: 320 contacts; optimized: 515 contacts), suggesting broader pocket engagement in this representative docked configuration. The optimized ligand also displays a richer polar-contact pattern, with nine hydrogen bonds detected under the same ChimeraX settings. These observations provide qualitative structural context for the predicted affinity–property trend and are consistent with plausible binding-site accommodation, but they remain descriptive and pose-dependent rather than confirmatory evidence of stronger binding or improved biological activity.

M^pro^ (baseline: cinanserin; optimized: CHEMBL4563204).

For M^pro^, both ligands occupy the same binding cleft with broadly similar pocket packing. The total contact counts are comparable under identical ChimeraX settings (baseline: 396 contacts; optimized: 385 contacts), suggesting similar overall burial and shape complementarity in the selected poses. The optimized ligand preserves the core anchoring region while displaying a slightly richer polar interaction pattern, with two hydrogen bonds, compared with one for the baseline. Thus, the pose analysis supports the cautious interpretation that the selected candidate can be accommodated in a plausible binding pose while maintaining similar pocket occupancy and modestly changing the displayed polar-contact pattern. Thus, the docking analysis should be interpreted as a structural plausibility check rather than validation of improved Mpro binding or antiviral activity.

### 4.4. EGFR Kinase Case Study with Experimentally Tested Compounds

To further test Nevermore in a target space with extensive experimental activity data, we evaluated the human epidermal growth factor receptor (EGFR; PubChem protein accession P00533) [55]. EGFR is a kinase-superfamily oncology target with many tested compounds and known inhibitors, making it useful for asking whether the database-grounded retrieval and ranking procedure behaves consistently with experimentally reported activity. In this case, the goal was not only to generate candidates with favorable predicted affinity–property trade-offs, but also to examine whether the retrieval geometry is aligned with known EGFR activity trends.

**Figure 8:**
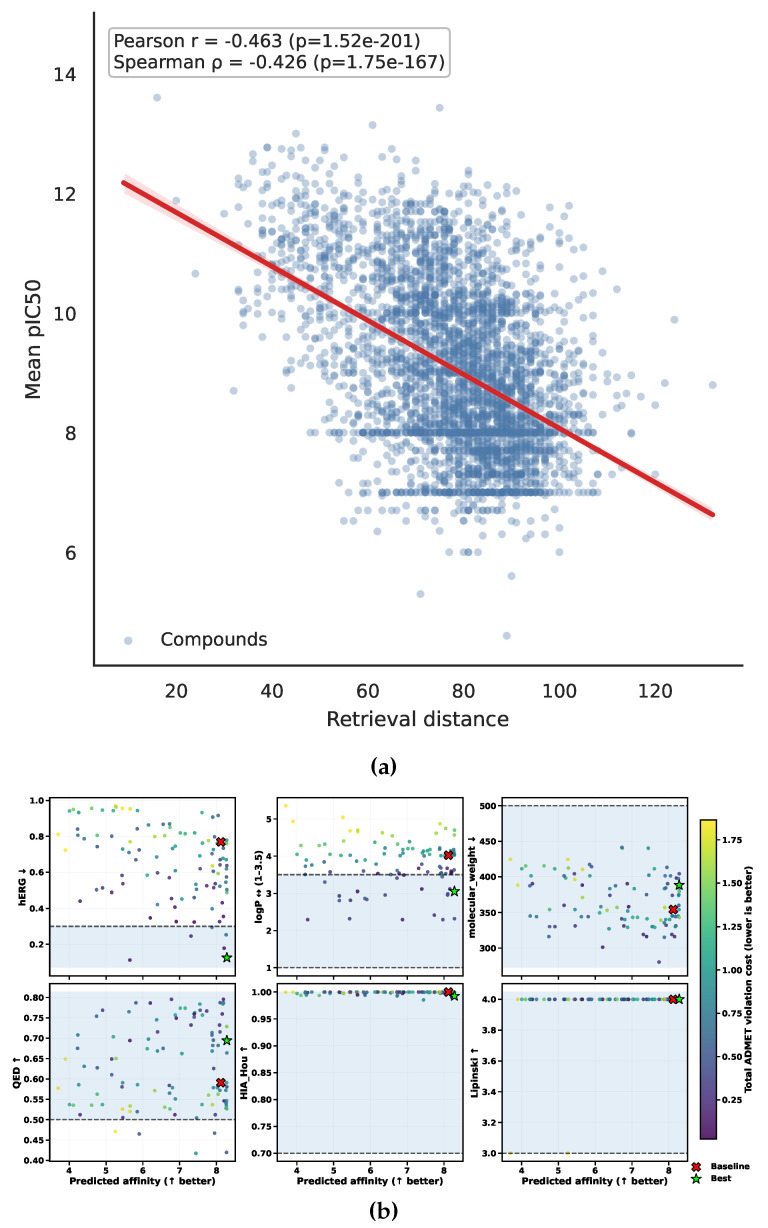
EGFR kinase case study. (**a**) Retrieval distance is negatively associated with experimental EGFR activity, indicating that closer compounds tend to have higher reported pIC_50_. (**b**) Pareto-aware prioritization of 100 retrieved EGFR candidates using predicted affinity and ADMET/property proxies. The baseline ligand is marked with a red ×, and the selected representative candidate is marked with a green star.

We first downloaded EGFR-tested compounds from PubChem and retained molecules with available structures and quantitative activity values. Activity values were converted to pIC_50_, where higher values indicate stronger reported activity. We then compared the Nevermore retrieval distance against mean pIC_50_ across the filtered EGFR compound set. These EGFR retrieval and prioritization results are summarized in Figure 8. Retrieval distance was negatively associated with experimental activity (Pearson r=−0.463, $p=1.52\times 10^{-201}$; Spearman ρ=−0.426, $p=1.75\times 10^{-167}$), indicating that compounds closer under the retrieval metric tend to have stronger reported EGFR activity. This provides an activity-based check that the retrieval distance captures biologically relevant structure in a well-studied kinase target space.

We next applied the multi-objective prioritization workflow to EGFR and evaluated the top-100 retrieved candidates using predicted affinity and ADMET/property proxies. The selected representative candidate was chosen from the affinity–property trade-off plot rather than by predicted affinity alone. This candidate remained in a high-predicted-affinity region while moving toward lower total property-violation cost, including improved hERG and molecular weight relative to the baseline. Thus, the EGFR result supports the use of Nevermore as a Pareto-aware candidate-prioritization workflow rather than an affinity-only ranking method.

The EGFR baseline compound was PubChem CID 5328234, and the selected optimized compound was PubChem CID 5328371. The two molecules share a related purine-like EGFR-relevant scaffold but differ in substituent patterns, indicating that the optimized candidate remains close to the original scaffold while shifting toward a better predicted affinity–property balance. As an additional known-drug neighborhood check, we compared both compounds against an OpenTargets/PubChem-derived set of EGFR-associated drugs with available SMILES. The optimized compound showed slightly higher average similarity to this known-drug set than the baseline compound (60.07 vs. 58.70). At a similarity threshold of ≥75, tarloxotinib was closer to the optimized compound than BPIQ-i (rank 32 vs. 42), whereas BPIQ-i was closer to the baseline compound and tarloxotinib did not appear in the corresponding baseline thresholded list. This suggests that the optimized EGFR candidate modestly shifts toward a tarloxotinib-like known-drug neighborhood while preserving the original purine-like scaffold.

## 5. Discussion

Nevermore is a target-conditioned, database-grounded framework for computational lead prioritization and refinement. The workflow couples (i) an affinity oracle built from pretrained protein language model embeddings and count-based Morgan fingerprints, (ii) derivative-free multi-objective optimization over sparse discrete fingerprint edits to balance predicted affinity with ADMET/property constraints, and (iii) projection of edited fingerprints back to real compounds through nearest-neighbor retrieval from a large compound library. By optimizing a target-specific score while remaining anchored to existing chemical space, Nevermore provides a practical computational strategy for reducing large compound libraries to smaller candidate sets with favorable predicted binding–property trade-offs for downstream biological and medicinal-chemistry review.

Conceptually, this design sits between deep QSAR/proteochemometric modeling and modern target-conditioned molecular optimization. Engineered ligand descriptors remain useful in data-limited settings, while pretrained protein representations provide a scalable way to condition predictions on the target sequence [12,16]. Count-based Morgan fingerprints are central to this design because they provide discrete, editable molecular features, which are more directly controllable than continuous latent embeddings [11]. This makes it possible to search over sparse feature edits while still tracing the optimization trajectory back to molecular motifs and retrieved candidate structures.

Compared with goal-directed de novo generation, including latent-space models, reinforcement learning, and graph-editing approaches, Nevermore prioritizes validity and practical accessibility by grounding optimization in a curated compound library. This design helps reduce common failure modes in which proxy optimization drifts toward unrealistic or difficult-to-interpret molecules, a challenge that has been widely observed in molecular design benchmarks [28,29,30,34]. At the same time, feature-space optimization is naturally compatible with black-box search and Pareto-style selection, which are especially useful when objectives such as predicted affinity, hERG liability, QED, molecular weight, logP, HIA, and Lipinski constraints compete with one another [10,35].

Empirically, the three target settings test different aspects of this design. Menin evaluates whether Nevermore can improve predicted affinity–property trade-offs for an oncology-relevant protein-interaction target starting from a clinically optimized inhibitor. SARS-CoV-2 M^pro^ tests the same workflow in a viral protease setting, where the baseline ligand already has strong predicted affinity but less favorable property proxies. EGFR adds a kinase-superfamily oncology case with many experimentally tested compounds, allowing us to examine whether the retrieval geometry is consistent with known activity trends rather than only model-predicted scores. In this setting, lower retrieval distance was associated with higher reported pIC_50_. Together, these results suggest that Nevermore does not simply optimize an abstract fingerprint vector, but can steer retrieval toward real molecules with biologically and chemically interpretable behavior across protein-interaction, protease, and kinase target contexts.

From a practical drug-discovery perspective, these optimization outcomes are most meaningful as a pre-experimental triage step rather than as final evidence of activity. For menin, the observed shift toward higher predicted affinity with improved property proxies suggests that Nevermore may help identify chemically reviewable alternatives around a clinically relevant protein–protein interaction target, where follow-up would require menin–KMT2A displacement or target-engagement assays. For SARS-CoV-2 M^pro^, the main biological value is different: the baseline ligand already has high predicted affinity, so the useful outcome is the identification of retrieved compounds that maintain plausible predicted protease compatibility while reducing property liabilities such as hERG risk or unfavorable drug-likeness. For EGFR, the association between retrieval distance and reported pIC_50_ provides an external activity-based check that the retrieval geometry remains connected to experimentally characterized kinase chemical space. Together, these results suggest that Nevermore can reduce a large compound library to a smaller set of real, target-relevant molecules for medicinal-chemistry review, orthogonal computational assessment, and biochemical or cellular validation.

A key advantage of Nevermore is that the optimization path is traceable. Because the optimizer proposes sparse edits in fingerprint space and the final candidates are retrieved from real molecules, the selected compounds can be examined at multiple levels: objective-level trade-offs, edited bucket sets, bucket-to-fragment mappings, and qualitative binding-pose inspection. This provides a useful bridge between numerical optimization and medicinal-chemistry interpretation. However, these interpretations should be viewed as hypothesis-generating rather than mechanistic proof. Hashed Morgan fingerprints can collide, meaning that distinct local chemical environments may map to the same bucket. Therefore, an edited bucket may correspond to multiple possible structural explanations. In our workflow, this ambiguity is partially mitigated by projecting edited fingerprints back to concrete library molecules, allowing the proposed edits to be inspected in chemically valid candidate structures. Recent work on reverse engineering ECFP-style fingerprints into molecular structures suggests a complementary future direction in which optimized fingerprints could be decoded beyond nearest-neighbor retrieval [56].

The main limitations of the present study are related to validation, library coverage, predictor calibration, and benchmarking scope. First, the reported improvements are based on predicted affinity values, ADMET/property proxies, retrieved library molecules, and qualitative pose inspection. These analyses are useful for computational prioritization, but they do not replace biochemical binding assays, cellular assays, pharmacokinetic evaluation, toxicity testing, or prospective synthesis. Second, the attainable candidate quality depends on the coverage of the retrieval library. If the library does not contain molecules near a beneficial edited fingerprint, nearest-neighbor projection may weaken or redirect the intended optimization step. Third, the affinity and ADMET models may be less reliable for targets or chemical series that are poorly represented in the training data, so uncertainty-aware scoring and calibration analysis will be important for future development. Finally, the current baselines are intentionally simple and interpretable. Random sampling and similarity retrieval are appropriate controls for this proof-of-concept study, but broader benchmarking against active-learning prioritization, Bayesian optimization, generative design, and additional target-conditioned molecular optimization workflows will be needed before making stronger claims of general superiority.

Overall, Nevermore demonstrates that database-grounded fingerprint steering can provide a controllable, inspectable, and hypothesis-generating route for target-conditioned computational candidate prioritization. Rather than replacing experimental lead optimization, the framework is best viewed as a hypothesis-generation and triage tool: it identifies retrieved molecules with favorable predicted affinity–property trade-offs, highlights the fingerprint edits associated with those trade-offs, and provides candidate sets for follow-up computational, medicinal-chemistry, and experimental evaluation.

## 6. Conclusions

Nevermore provides a database-grounded framework for target-conditioned computational lead prioritization using sparse fingerprint edits, derivative-free multi-objective optimization, and nearest-neighbor retrieval from large compound libraries. The main contribution is methodological. The framework turns QSAR-style molecular descriptors into an editable search space while maintaining chemical validity by projecting optimized fingerprints back to real molecules. Across menin, SARS-CoV-2 M^pro^, and EGFR, the results show favorable predicted affinity–property trade-offs relative to random sampling, baseline-centered similarity retrieval, and embedding-based retrieval. The EGFR case further links retrieval distance with reported pIC_50_ trends in a kinase target space.

The framework is most useful as a hypothesis-generation and triage tool rather than as a replacement for experimental lead optimization. Its traceability allows users to inspect edited fingerprint dimensions, associated fragment-level hypotheses, and, where available, qualitative representative binding poses, providing a bridge between numerical optimization and medicinal-chemistry review. At the same time, performance depends on the coverage of the retrieval library, the calibration of the affinity and ADMET/property predictors, and the ambiguity introduced by hashed fingerprint collisions. In addition, the use of 2D count-based Morgan fingerprints may introduce representation bias, since these descriptors do not fully capture stereochemistry, conformational flexibility, or target-specific 3D interaction geometry. Future work should incorporate uncertainty-aware scoring, collision-aware and stereochemistry-aware representations, richer 3D descriptors or pose-conditioned features, broader benchmarking, and prospective experimental feedback. Together, these directions can further strengthen Nevermore as a practical component of iterative, data-guided lead refinement workflows.

## Figures and Tables

**Table 1:** Comparison on BIOSNAP, BindingDB, and DAVIS datasets. Mean ± s.e.m. over 5 random seeds. The Kruskal–Wallis (KW) *p*-values are from the approximate sensitivity analysis for AUPR/AUROC. Bold indicates the best value.

Benchmark	Model	AUPR	AUROC	KW *p* (AUPR/AUROC)
BIOSNAP (19,238)	Firm–DTI (MolE)	**0.919 ± 0.001**	**0.910 ± 0.001**	0.0172/0.0103
	Nevermore–DTI (Morgan FP)	0.890 ± 0.006	0.891 ± 0.006	
	MolTrans [19]	0.885 ± 0.005	0.876 ± 0.007	
	GNN-CPI [18]	0.890 ± 0.004	0.879 ± 0.007	
	DeepConv-DTI [17]	0.889 ± 0.005	0.883 ± 0.002	
BindingDB (12,668)	Firm–DTI (MolE)	**0.647 ± 0.001**	**0.916 ± 0.001**	0.0183/0.1545
	Nevermore–DTI (Morgan FP)	0.591 ± 0.007	0.903 ± 0.008	
	MolTrans [19]	0.598 ± 0.013	0.898 ± 0.009	
	GNN-CPI [18]	0.578 ± 0.015	0.900 ± 0.004	
	DeepConv-DTI [17]	0.611 ± 0.015	0.908 ± 0.004	
DAVIS (2086)	Firm–DTI (MolE)	**0.454 ± 0.001**	0.870 ±0.001	0.0006/0.0011
	Nevermore–DTI (Morgan FP)	0.437 ± 0.003	**0.920 ± 0.002**	
	MolTrans [19]	0.335 ± 0.017	0.889 ± 0.007	
	GNN-CPI [18]	0.269 ± 0.020	0.840 ± 0.012	
	DeepConv-DTI [17]	0.299 ± 0.039	0.884 ± 0.008	

**Table 2:** Menin and SARS-CoV-2 M^pro^ candidate-set summary (N=100). Mean ± std over generated candidates (excluding baseline). Best per target is bold.

Method	Affinity↑	hERG↓	QED↑	MW↓
**SARS-CoV-2 M^pro^**				
Baseline	8.266	0.939	0.440	340.492
Random	7.098 ± 1.299	0.614 ± 0.285	0.526 ± 0.230	462.960 ± 453.090
RDKFP+Tversky	7.229 ± 0.839	0.500 ± 0.231	0.676 ± 0.203	301.715 ± 47.191
MolE	7.268 ± 1.167	0.933 ± 0.045	0.668 ± 0.116	335.606 ± 32.857
Nevermore	**7.283 ± 1.064**	**0.411 ± 0.274**	**0.740 ± 0.179**	**292.959 ± 34.844**
**Menin**				
Baseline	5.765	0.945	0.382	630.831
Random	6.056 ± 1.362	0.858 ± 0.113	0.623 ± 0.179	449.249 ± 104.242
RDKFP+Tversky	6.934 ± 0.998	0.625 ± 0.284	0.545 ± 0.220	408.422 ± 128.816
MolE	6.429 ± 0.826	0.919 ± 0.068	0.477 ± 0.115	541.895 ± 74.463
Nevermore	**7.060 ± 0.707**	**0.454 ± 0.230**	**0.712 ± 0.154**	**319.844 ± 72.276**

**Figure A1:**
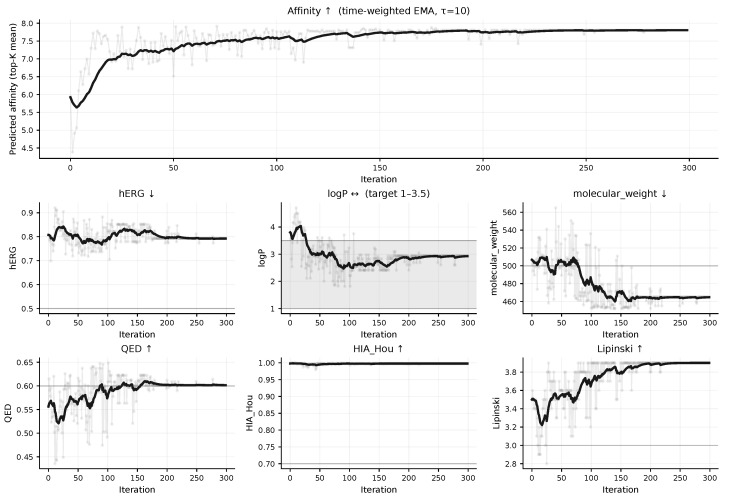
Nevermore optimization trajectories over 300 iterations. (**Top**): Predicted affinity statistic per iteration. (**Bottom**): Corresponding ADMET endpoints. Light traces show raw per-iteration values and bold curves show a time–weighted EMA to emphasize the trend.

**Figure A2:**
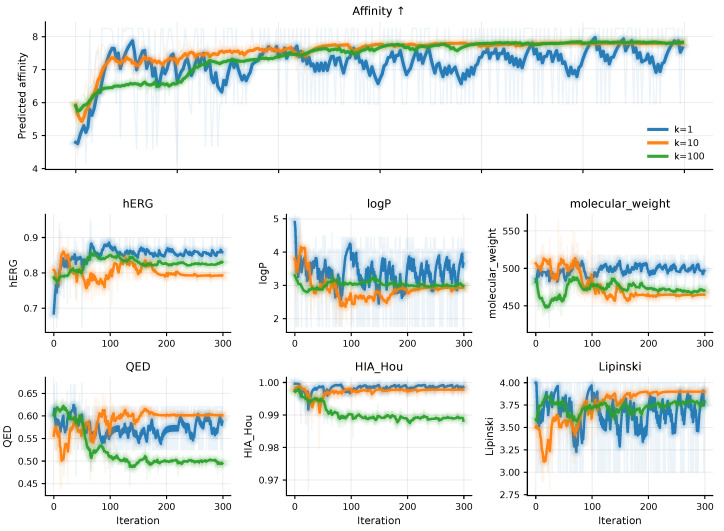
Sensitivity to retrieval neighborhood size *K*. Optimization trajectories for predicted affinity and developability proxies under different retrieval neighborhood sizes (K∈{1,10,100}). Small *K* yields higher-variance trajectories because discrete fingerprint edits can abruptly change the nearest-neighbor identity, producing noisy objective feedback. Large *K* smooths trajectories by aggregating over a broader neighborhood, but can weaken the optimization signal by diluting the effect of sparse integer edits. We therefore use K=10 as a practical trade-off that stabilizes optimization while preserving responsiveness to steering.

## Data Availability

All code for Nevermore, including training, optimization, retrieval, evaluation scripts, preprocessing scripts, random-seed settings, and experiment configurations, is publicly available at https://github.com/EESI/nevermore (accessed on 17 June 2026). The benchmark datasets used in this study are publicly available. We obtained the BindingDB Patent and DTI-DG tasks through the Therapeutics Data Commons (TDC) at https://tdcommons.ai/ (accessed on 17 June 2026). For the standard in-domain DTI benchmarks, including DAVIS and BindingDB, and their preprocessing/binarization used in MolTrans-style evaluations, we followed the publicly released dataset resources at https://github.com/kexinhuang12345/MolTrans/tree/master/dataset (accessed on 17 June 2026). The ADMET/property annotations used for screening were generated using ADMET-AI, available at https://github.com/swansonk14/admet_ai (accessed on 17 June 2026); the generated ADMET-AI reports and property tables used in this study are provided with the Nevermore repository. Nevergrad was used for derivative-free optimization and is publicly available at https://github.com/facebookresearch/nevergrad (accessed on 17 June 2026). Any additional preprocessing scripts and experiment configurations used in this work are provided in our repository.

## Appendix A. Supplementary Terminology and Practical Software Use

**Terminology.** To make the workflow more accessible to readers from different backgrounds, we summarize the main technical terms used in this manuscript.

- **Target-conditioned model:** A model whose prediction depends on the specific protein target. In this work, the same ligand may receive different predicted affinity scores depending on the protein sequence provided as input.
- **Affinity oracle:** The frozen target-conditioned protein–ligand affinity model queried during optimization. The oracle predicts affinity scores for candidate molecules but is not retrained during the molecular search.
- **Geometry-aware model:** A model that represents proteins and ligands in a shared embedding space and uses their relative distance or similarity to estimate binding affinity. In this setting, protein–ligand pairs predicted to interact strongly are encouraged to be closer in the learned space, while weaker or mismatched pairs are placed farther apart.
- **Morgan fingerprint:** A fixed-length numerical representation of a molecule that summarizes local chemical environments around atoms. Morgan fingerprints are commonly used in cheminformatics because they provide an efficient and interpretable way to compare molecules.
- **Morgan fingerprint bucket:** One coordinate of a hashed count-based Morgan fingerprint. Changing a bucket count can encourage or discourage certain local chemical environments, although hashing can map multiple chemical environments to the same bucket.
- **Sparse edit:** A small change applied to only a limited subset of fingerprint buckets. Sparse edits make the optimization easier to inspect because only a few molecular features are changed at a time.
- **Fingerprint steering:** The process of guiding molecular search by editing fingerprint features rather than directly generating a new chemical structure.
- **Derivative-free optimization:** An optimization strategy that does not require gradients. This is useful here because the workflow includes discrete fingerprint edits and nearest-neighbor retrieval steps that are not easily differentiable.
- **Projection:** The step that maps an edited fingerprint back to nearby real molecules in a retrieval library. This ensures that the final reported candidates are valid database molecules rather than unconstrained numerical vectors.
- **Database-grounded retrieval:** The process of selecting real molecules from a reference compound library, such as a ChEMBL-derived library, instead of relying on fully de novo molecule generation.
- **ADMET/property proxies:** Predicted or computed quantities related to absorption, distribution, metabolism, excretion, toxicity, and drug-likeness. These values are used as inexpensive screening signals to flag potential developability liabilities, not as experimentally validated safety measurements.
- **Pareto-aware optimization:** An approach that considers trade-offs among multiple competing objectives rather than optimizing only one score. For example, a molecule with the best predicted affinity may not be preferred if it has poor drug-like properties.
- **Pareto-style candidate:** A compound selected because it offers a favorable balance between competing objectives, such as predicted affinity, toxicity-related proxies, molecular weight, logP, QED, or other property constraints.

**Software workflow.** The public implementation is available at https://github.com/EESI/nevermore (accessed on 17 June 2026). The repository is organized as a staged command-line workflow consisting of data ingest, feature building, optimization, retrieval, optional visualization, optional docking, and report generation. The main configuration file is configs/default.yaml. The most important user-facing settings are the target protein sequence, baseline ligand SMILES, model checkpoint, compute device, number of editable ligand buckets, optimization budget, ADMET penalty weight β, retrieval-library path, number of reported candidates, and writable output directory.

A minimal command-line workflow is shown below.

The retrieval command runs the core pipeline through candidate retrieval and produces outputs such as an optimized edit summary, a target manifest, and candidate_ligands.csv. The report command continues to consolidated outputs such as nevermore_report.csv. Each stage writes cached artifacts under the configured output directory using a short signature, so repeated runs with unchanged inputs and configuration can reuse previous outputs. Optional docking requires enabling docking in the configuration and providing the receptor PDBQT file and docking box.Optional ADMET scoring requires the corresponding ADMET-AI package, available at https://github.com/swansonk14/admet_ai (accessed on 17 June 2026).

## Appendix B. Dataset Statistics

We evaluate the DTI prediction component using four widely used benchmarks: DAVIS, BindingDB, BIOSNAP (ChG-Miner), and TDC DTI-DG [39,43,44,45]. DAVIS, BindingDB, and BIOSNAP follow the processed in-domain splits used in prior DTI studies, including MolTrans [19]. These datasets are used for binary DTI prediction, with AUROC and AUPR as the main classification metrics.

For BIOSNAP, the original dataset contains known positive drug–target interactions [45]. Following prior practice, negative examples are sampled from unobserved drug–target pairs, producing a balanced positive/negative dataset. For DAVIS and BindingDB [43,44], experimentally measured binding affinities are converted into binary labels using the standard threshold $K_{d}<30$, where pairs below this threshold are treated as positive interactions. To maintain balanced training, the same number of negative pairs as positive pairs is sampled for the training set, while the original negative ratios are retained in the validation and test sets [39].

In addition to these in-domain benchmarks, we include TDC DTI-DG, a temporal out-of-distribution benchmark derived from BindingDB drug–target interactions, with patent-year information [39]. In this benchmark, each domain corresponds to DTIs patented in a specific year. Models are trained on interactions patented from 2013 to 2018 and evaluated on later interactions from 2019 to 2021, simulating a realistic prospective generalization setting. TDC DTI-DG is treated as an affinity prediction task and evaluated using the Pearson correlation coefficient (PCC). In our pipeline, the model trained on this benchmark is also used as the frozen affinity-scoring oracle for constrained multi-objective optimization.

**Table A1:** Dataset split sizes used for training, validation, and evaluation.

Dataset	Train	Val	Test	Task
DAVIS [19]	2086	3006	6017	Binary DTI classification
BindingDB [19]	12,668	6484	13,289	Binary DTI classification
BIOSNAP [19]	19,238	2748	5497	Binary DTI classification
TDC DTI-DG [39]	146,479	36,539	49,028	Temporal affinity prediction

## Appendix C. Training Details

We trained our model (Nevermore–DTI) on a single NVIDIA H100 GPU with 80 GiB of memory. The total number of parameters is ≈$3.5\times 10^{9}$, with the vast majority residing in the pretrained encoders. During training, we update only ∼9 million parameters, while keeping the pretrained encoders frozen. For the protein encoder, we use the ESM2 model esm2_t12_35M_UR50D from Facebook AI’s repository https://huggingface.co/facebook/esm2_t12_35M_UR50D (accessed on 17 June 2026).

We optimize with AdamW ($\epsilon =10^{-6}$) and use a cosine learning-rate schedule with linear warmup. The main hyperparameters are summarized in Table A2.

**Table A2:** Training hyperparameters.

Hyperparameter	Value
Batch size	20
Learning rate	$5\times 10^{-5}$ (cosine decay; 500 warmup steps)
Optimizer	AdamW, $\epsilon =10^{-6}$
Weight decay	0.1
Triplet margin α	0.9
Max protein length	1500 residues
Epochs	40
Total parameters	≈$3.5\times 10^{9}$
Trainable parameters	∼$9\times 10^{6}$
GPU used	NVIDIA H100 80 GiB
Training time	∼40 h per run

## Appendix D. Morgan Fingerprint Size Ablation

To isolate the impact of ligand featurization, we train our model with Morgan fingerprints at multiple bit-lengths. Table A3 reports the mean ± std PCC across five random seeds (best checkpoint per seed). Among the evaluated sizes, 2048-bit fingerprints provide the best average performance. We therefore adopt this setting as the default Morgan configuration for comparisons and downstream analyses, unless otherwise stated.

**Table A3:** Effect of Morgan fingerprint bit-length on BindingDB Patent (Pearson correlation, PCC). Results are mean ± std across 5 random seeds.

Morgan FP Size (Bits)	PCC (Mean ± Std)
1024	0.5710 ± 0.0075
2048	0.5701 ± 0.0091
4096	0.5627 ± 0.0020

## Appendix E. ADMET Profile Under Different Morgan Fingerprint Sizes

To check whether the Morgan fingerprint resolution materially changes the physicochemical profile of retrieved ligands, we summarize the major predicted ADMET properties for the final retrieved candidate sets under each fingerprint size. Table A4 reports mean ± std for molecular weight (MW), logP, HIA, hERG, QED, and Lipinski.

**Table A4:** Major predicted ADMET properties (mean ± std) for retrieved candidates under different Morgan fingerprint sizes.

Morgan FP Size (Bits)	MW	logP	HIA	hERG	QED	Lipinski
1024	429.8 ± 44.8	3.46 ± 1.20	0.992 ± 0.018	0.776 ± 0.149	0.579 ± 0.147	3.69 ± 0.45
2048	465.9 ± 38.5	3.63 ± 1.10	0.997 ± 0.005	0.803 ± 0.141	0.598 ± 0.115	3.67 ± 0.50
4096	454.9 ± 47.9	3.09 ± 1.05	0.991 ± 0.022	0.799 ± 0.144	0.578 ± 0.119	3.65 ± 0.44

## Appendix F. Multi-Objective Focus: Affinity with Property Preservation

In our multi-objective setting, the primary objective is affinity, i.e., we aim to increase predicted binding strength while remaining on the empirical molecule manifold. Nevermore therefore emphasizes affinity gains, but not by drifting into unrealistic edits. Two mechanisms keep the search property-aware: (i) a weighted ADMET penalty that discourages liability-increasing steps, and (ii) the projection step that maps each edited fingerprint back to a top-*K* neighborhood of real database molecules. Together, these constraints act as an invariance regularizer, yielding affinity improvements that remain consistent with desirable ADMET behavior. Figure A1 illustrates this behavior over 300 iterations: affinity increases while key endpoints remain stable or improve, with the exponential moving average (EMA) curve highlighting the underlying trend beneath projection-induced iteration-to-iteration jitter.

## Appendix G. Sensitivity to Retrieval Neighborhood Size K

Figure A2 reports optimization trajectories for predicted affinity and developability proxies under different retrieval neighborhood sizes (K∈{1,10,100}). Small neighborhoods, such as K=1, produce higher-variance trajectories because sparse fingerprint edits can abruptly change the identity of the single nearest retrieved molecule, leading to noisy objective feedback. In contrast, large neighborhoods, such as K=100, smooth the trajectories by averaging over a broader set of retrieved compounds, but can weaken the effect of each edit by diluting the local optimization signal. We therefore use K=10 as a practical trade-off that stabilizes projection while preserving responsiveness to sparse fingerprint steering.

## Appendix H. Additional Optimization Success Metrics

To further clarify the optimization outcomes, we report two complementary summaries. Table A5 shows the Pareto-aware threshold-based selection results, where candidates are counted as successful only if they pass predefined affinity and ADMET/property thresholds. This is a strict selection view. For menin, the baseline molecule had a high predicted hERG risk, so the hERG threshold became the main bottleneck. As a result, only a small number of candidates passed the full ADMET criteria when hERG was included, even though a larger fraction passed the non-hERG ADMET criteria.

Table A6 provides a baseline-relative view of the same optimization results. This table shows that, although the strict hERG cutoff limited the number of selected menin candidates in Table A5, many optimized candidates still moved in the desired direction relative to the toxic baseline molecule. Thus, the optimization improved the candidate pool, even when many molecules did not fully satisfy the final strict selection threshold.

For SARS-CoV-2 Mpro, the baseline molecule already had a high predicted affinity. Therefore, fewer candidates were expected to exceed the baseline affinity, because the starting point was already strong. In this case, the value of the optimization is better reflected by the ADMET and property improvements, while maintaining competitive predicted affinity.

Together, these two tables separate strict final candidate selection from directional optimization improvement. The first table shows which candidates satisfied the final selection thresholds, while the second table shows whether the optimization moved candidates toward more favorable affinity and developability profiles.

**Table A5:** Pareto-aware threshold-based summary of optimization success for the 100 retrieved candidates per target. The affinity objective is defined as predicted affinity greater than or equal to the target-specific baseline predicted affinity. Each column reports the number and percentage of candidates passing the corresponding selection threshold.

Target	All ADMET Include hERG	All ADMET Without hERG	hERG x≤0.4	logP 1≤x≤3.5	MW x≤500	QED x≥0.5	HIA_Hou x≥0.7	Lipinski x≥3.0
Menin	2 (2.0%)	31 (31.0%)	3 (3.0%)	72 (72.0%)	54 (54.0%)	65 (65.0%)	100 (100.0%)	95 (95.0%)
SARS-CoV-2 Mpro	27 (27.0%)	56 (56.0%)	55 (55.0%)	65 (65.0%)	100 (100.0%)	84 (84.0%)	98 (98.0%)	100 (100.0%)

**Table A6:** Baseline-relative improvement summary for the 100 optimized candidates per target. For each objective, the table reports the baseline value, the number and percentage of optimized candidates that improved relative to the baseline or satisfied the desired range, and the best optimized value observed among the retrieved candidates.

Objective	Improvement Rule	Menin			SARS-CoV-2 Mpro		
		Baseline	Improved Candidates	Best Optimized	Baseline	Improved Candidates	Best Optimized
Predicted affinity	x> baseline	5.765	69 (69.0%)	8.225	8.266	5 (5.0%)	8.283
hERG	x< baseline	0.945	91 (91.0%)	0.356	0.939	96 (96.0%)	0.019
logP	|x−2.25|<|baseline−2.25|	4.679	91 (91.0%)	2.242	4.382	90 (90.0%)	2.251
Molecular weight	x< baseline	630.831	100 (100.0%)	441.919	340.492	91 (91.0%)	238.718
QED	x> baseline	0.382	88 (88.0%)	0.743	0.440	91 (91.0%)	0.929
HIA_Hou	x> baseline	0.999	31 (31.0%)	1.000	0.974	93 (93.0%)	1.000
Lipinski	3.0≤x≤5.0	3.000	95 (95.0%)	4.000	4.000	100 (100.0%)	4.000

## Appendix I. Docking Settings for Qualitative Pose Visualization

Docking was performed with AutoDock Vina using exhaustiveness 16 and 10 output modes. The docking settings used for the qualitative pose visualizations are summarized below.

**Table A7:** Docking settings used for qualitative pose visualization.

Target	Docking Box Center	Docking Box Size
SARS-CoV-2 M^pro^	(−6,−3,−16)	20×20×20
Menin	(15,−64,1)	34×34×34
